# Trends of malaria infection in pregnancy in Ghana over the past two decades: a review

**DOI:** 10.1186/s12936-021-04031-3

**Published:** 2022-01-04

**Authors:** Joseph Osarfo, Gifty Dufie Ampofo, Harry Tagbor

**Affiliations:** Department of Community Medicine, School of Medicine, University of Health and Allied Sciences, Ho, Volta Region Ghana

**Keywords:** *Plasmodium falciparum*, Malaria in pregnancy, Asymptomatic parasitaemia, Prevalence, Trends, Ghana

## Abstract

**Background:**

There has been a global decline in malaria transmission over the past decade. However, not much is known of the impact of this observation on the burden of malaria infection in pregnancy in endemic regions including Ghana. A narrative review was undertaken to help describe trends in malaria infection in pregnancy in Ghana. Among others, such information is important in showing any progress made in malaria in pregnancy control.

**Methods:**

To describe trends in pregnancy-associated malaria infection in Ghana, a search and review of literature reporting data on the prevalence of asymptomatic *Plasmodium falciparum* infection in pregnancy was conducted.

**Results:**

Thirty-six (36) studies, conducted over 1994–2019, were included in the review. In the northern savannah zone with largely seasonal malaria transmission, prevalence appeared to reduce from about 50–60% in 1994–2010 to 13–26% by 2019. In the middle transitional/forest zone, where transmission is perennial with peaks in the rainy season, prevalence apparently reduced from 60% in the late 1990 s to about 5–20% by 2018. In the coastal savannah area, there was apparent reduction from 28 to 35% in 2003–2010 to 5–11% by 2018–2019. The burden of malaria infection in pregnancy continues to be highest among teenagers and younger-aged pregnant women and paucigravidae.

**Conclusions:**

There appears to be a decline in asymptomatic parasite prevalence in pregnancy in Ghana though this has not been uniform across the different transmission zones. The greatest declines were noticeably in urban settings. Submicroscopic parasitaemia remains a challenge for control efforts. Further studies are needed to evaluate the impact of the reduced parasite prevalence on maternal anaemia and low birthweight and to assess the local burden of submicroscopic parasitaemia in relation to pregnancy outcomes.

**Supplementary Information:**

The online version contains supplementary material available at 10.1186/s12936-021-04031-3.

## Background

Malaria in pregnancy (MIP) remains a public health burden in endemic areas. Adverse outcomes include maternal anaemia and mortality, fetal growth restriction, abortion, preterm delivery and low birthweight following parasite placental sequestration and subsequent immunological processes [1–4]. *Plasmodium falciparum* infection accounts for the greater burden of morbidity and mortality though *Plasmodium vivax* may also be important in Southeast Asia and South America [5, 6]. Over 11 million pregnant women in sub-Saharan Africa were exposed to malaria infection during their pregnancy in 2019 and 16% of low birthweight cases were attributable to malaria [7].

Insecticide-treated bed nets (ITNs) and intermittent preventive treatment with sulfadoxine–pyrimethamine (IPTp-SP) are the key preventive interventions in endemic areas with stable transmission. Use of ITNs has been reported to underlie a 23% reduction in placental parasitaemia in a systematic review while ≥ 3 doses of IPTp-SP has been associated with up to 56% reduction in the risk of peripheral parasitaemia and a significant reduction in submicroscopic infections [8–10].

In Ghana, MIP accounted for 17.6% of outpatient department attendance, 13.7% of hospital admissions among pregnant women and 3.4% of maternal deaths in 2014 with the first two indicators declining to 14% and 11% in 2015 [11, 12]. There has been an appreciable decline in malaria transmission globally [7] but it remains unclear how this has impacted on the burden of malaria in pregnancy. Specifically, very little is known of trends in asymptomatic *P. falciparum* parasitaemia in pregnant women in Ghana and other endemic areas in spite of their vulnerability to malaria. Such data is important to help evaluate, directly or indirectly, the effectiveness of many years of targeted preventive interventions including IPTp-SP and ITNs. It will also be useful to identify where greater burdens persist for intensification of control interventions.

**Fig. 1:**
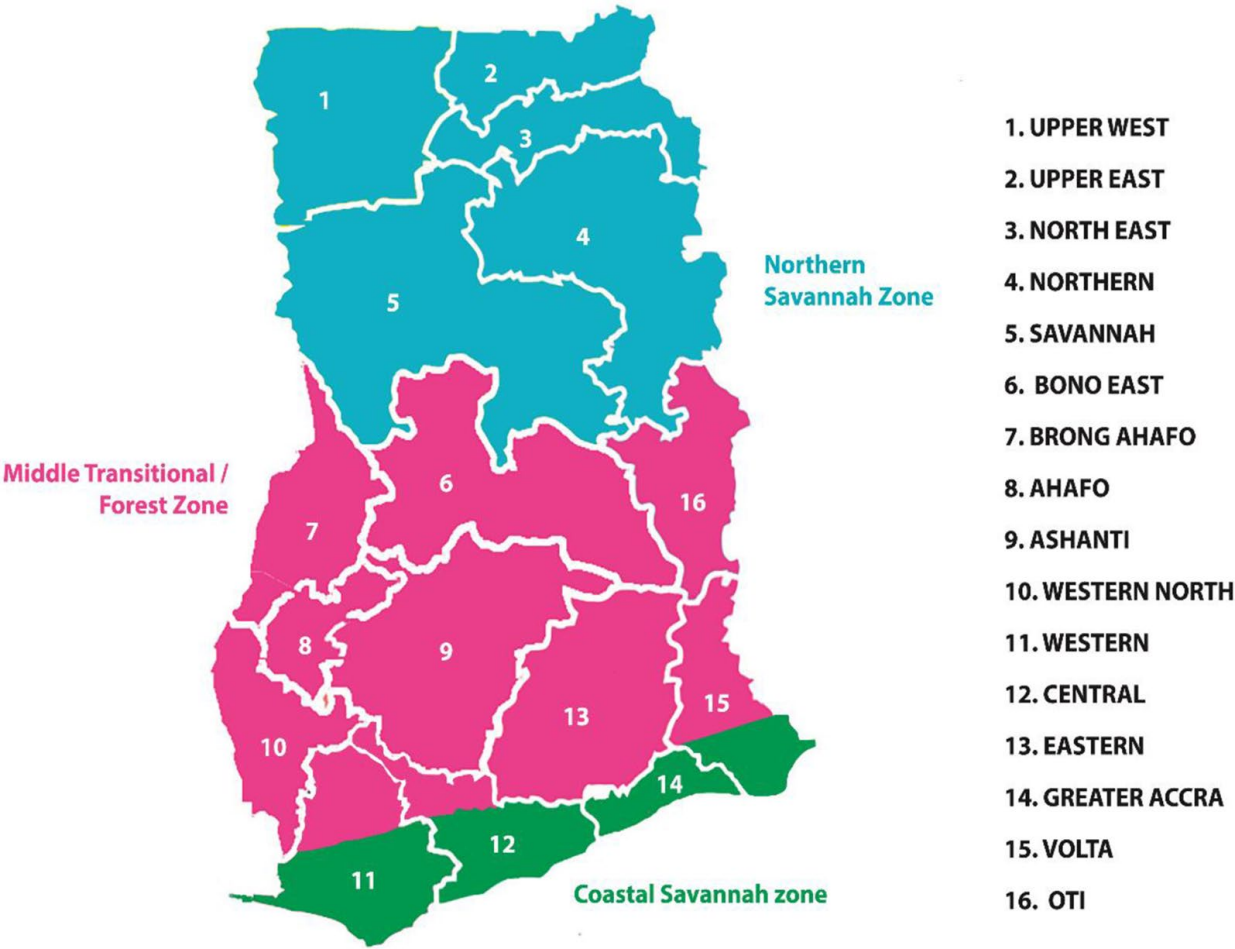
Map of Ghana showing the malaria epidemiological zones and the administrative regions (Source: Awine et al. [13])

This narrative review seeks to describe trends in asymptomatic parasitaemia prevalence among pregnant women in the three malaria ecological zones in Ghana; namely the northern savannah zone characterized by intense and seasonal transmission (with pockets of perennial transmission where irrigation projects are found), the transitional savannah/forest zone in the middle belt of the country with perennial and intense transmission and the coastal savannah belt [13] (see Fig. 1).

## Methods

To identify studies reporting prevalence of asymptomatic *P. falciparum* infection in Ghanaian pregnant women, a search was conducted in Google Scholar and PubMed using the terms (“*Plasmodium falciparum* parasitaemia in pregn*” OR “malaria infection in pregnancy) AND prevalence AND Ghana. The literature search was conducted from January to March, 2021 and was not restricted to any particular year or period of publication. Studies were included in the review if they reported prevalence of asymptomatic *P. falciparum* parasitaemia in women in Ghana anytime during pregnancy or at delivery and at any time of the year, if they were primary studies and also if they reported the period of data/sample collection. Titles/abstracts were screened and those deemed relevant were retrieved and further assessed for eligibility after checks for duplication. Data on parasite prevalence, method of detection, period over which study was conducted and whether peripheral/placental blood or placental biopsy was used were extracted from the included studies using a predesigned data extraction sheet. Parasite prevalence was compared over the years to describe trends.

Supporting data on malaria test positivity rates (number of parasitologically confirmed cases/total of suspected cases tested) among pregnant women was sourced from the district health information management system (DHIMS 2) database. The DHIMS 2 is an electronic database employed by the Ghana Health Service for reporting disease morbidity and mortality and receives monthly inputs from health facilities across the country. Suspected and parasitologically-confirmed MIP cases for Greater Accra, Ashanti and the former Northern region (now divided into Northern, North East and Savannah regions) over a 7-year period (2014–2020) were available to the authors and used to calculate test positivity rates for the respective years. The listed regions were chosen to represent the three malaria transmission settings (see Fig. 1).

## Results

### Selection of studies for inclusion in the review

**Fig. 2:**
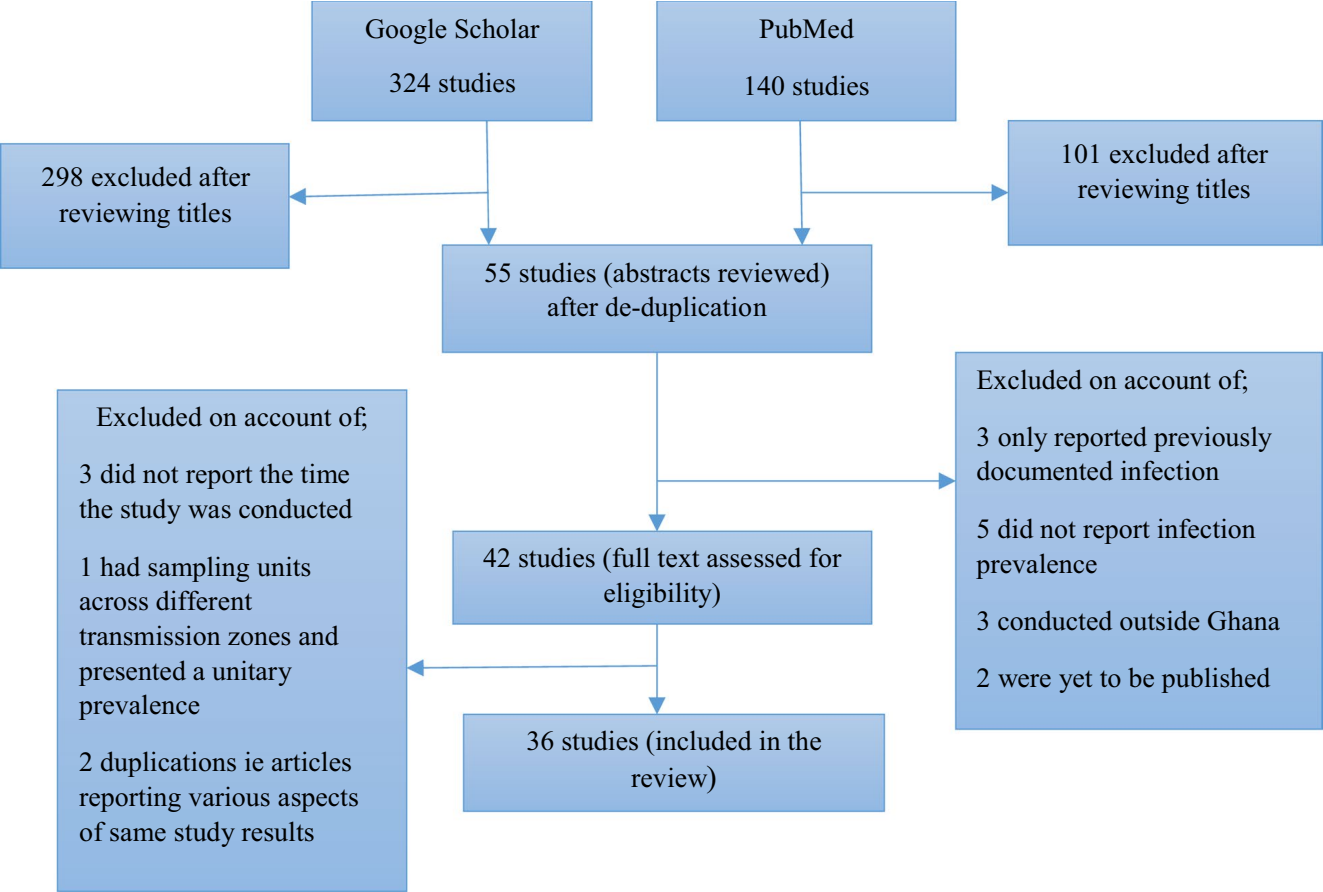
Flow diagram for literature search strategy

The search identified 464 studies (see Fig. 2). Of these, 422 were rejected for various reasons including not being deemed relevant on title/abstract review, duplications and lack of peer review. Of the 42 studies for which full texts were retrieved, 3 were further rejected for not indicating when they were conducted, 1 was rejected for having sampling units across different epidemiological zones and reporting a unitary prevalence and 2 more were rejected for duplication of data from studies already included. Finally, 36 studies comprising 34 published articles and 2 postgraduate (MPH) dissertations were included in the review (Table 1). The period of data/sample collection for the included studies, which is of key interest to the description of trends of malaria infection in pregnancy, covered 1994–2019. The articles were, however, published between 2000 and 2021. There were 9 studies conducted in the northern savannah zone, 14 covering the coastal savannah belt and 13 in the middle transitional/forest zone. Twenty-six of the studies utilized peripheral blood alone while 4 and 6 studies respectively used placental blood/tissue alone or combined peripheral and placental blood/tissue. Regarding the method of parasitaemia detection, 21 studies used microscopy alone while 4 used rapid diagnostic tests (RDT) alone, 1 used polymerase chain reaction (PCR) alone and 6 used multiple methods. Trends of malaria infection in pregnancy prevalence are described for each transmission zone.

**Table 1:** Studies reviewed for prevalence of maternal *P. falciparum* parasitaemia in Ghana

Zone	Study	Period of data collection	Prevalence during pregnancy and method of detection	Prevalence at delivery and method of detection
Northern Savannah	Browne et al. [14]	July 1994–Apr 1995	60% by microscopy	
	Clerk et al. [15]	Jun 2004–July 2006	47% overall by microscopy (32% and 59% before and after the rainy season respectively)	
	van Spronsen et al. [20]	June 2010–August 2010		52% by microscopy (placental blood)
	Williams et al. [16]	31 May 2010–31 October 2011	58.6% detected by both microscopy or PCR (combined) 53.6% by RDT	22.2% by RDT (peripheral blood) 28.7% by placental histology (31.3% in primigravidae vs. 25.4% in secundigravidae)
	Ahenkorah et al. [19]	May 2013–May 2014	21.6% by microscopy	
	Anabire et al. [17]	Oct 2016–Feb 2017	14. 1% by RDT 13.4% by PCR	
	Agyemang et al. [10]	Sept 2016–Aug 2017	Overall 25.9% by microscopy at 36 weeks (35.8% prevalence of malaria in those with no SP, 30.8% prevalence among those who took 1–2 doses of IPTp-SP and about 17% among those who took ≥ 3 doses)	
	Tibambuyah et al. [18]	Nov 2017–April 2018	13.8% by microscopy	
	Mwin et al. [21]	Jan 2019–April 2019		7% by microscopy (placental blood)
Coastal Savannah	Ofori et al. [22]	Jan 2003–Jan 2004	19.7% by microscopy	35.7% by microscopy (placental blood)
	Stephens et al. [26]	July–August 2008	5% by microscopy	2.5% by microscopy (placental blood)
	Wilson et al. [23]	June–August 2009	28.4% overall by microscopy (15.3% among IPTp users vrs 44.7% among non-IPTp users)	
	Orish et al. [24]	Jan–Oct 2010	23% overall by microscopy (34.6% in adolescents and 21.3% in adult pregnant women)	
	Orish et al. [25]	Mar–Oct 2010	23.3% by microscopy	
	Volker et al. [27]	Oct 2011–Jan 2012	10.6% by microscopy	
	Tay et al. [28]	April–July 2012	16.5% by microscopy	
	Lamptey et al. [29]	Nov 2013– Sept 2014	16.4% prevalence of asexual parasitaemia by PCR 29.7% prevalence of submicroscopic gametocytes also by PCR	
	Quakyi et al. [34]	Dec 2015–May 2017	Among two ANC cohorts 3.5% and 3.8% by microscopy 8.9% and 9.4% by PCR 42.2% and 43.1% by ultrasensitive PCR	Among two delivery cohorts 2.4% and 0% by microscopy (peripheral blood) 4.4% and 3% by PCR (peripheral blood) 12.6% and 8.7% by ultrasensitive PCR (peripheral blood) 0.3% and 0% by microscopy (placental blood) 2.5% and 1.7% by PCR (placental blood) 6.9% and 3.7% by ultrasensitive PCR (placental blood)
	Kiptoo [33]	June 2016	5.5% by microscopy	
	Obri et al. [35]	Jan 2017–Dec 2017		48.1% active placental infections by histology
	Afutu et al. [31]	April–June 2017	10.1% by microscopy 13.1% by RDT 13.8% by PCR	
	Fondjo et al. [32]	July-–ug 2018	10.1–11.4% by microscopy (two cohorts in Accra and Tarkoradi)	
	Offei [30]	12th June 2019–2nd July 2019	11.1% by RDT	
Middle transitional/forest zone	Mockenhaupt et al. [38]	Nov–Dec, 1998	63% overall by microscopy and PCR	
	Glover-Amengor et al. [36]	Rainy season of year 2000	35.1% by microscopy	
	Mockenhaupt et al. [39]	Jan 2000–Jan 2001	34% by peripheral blood microscopy 53% by peripheral blood PCR 19% by an HRP2 assay	41% by placental blood microscopy 59% by placental blood PCR 35% using HRP2 assay on placental blood
	Tagbor et al. [46]	March 2003–December 2004	Overall RDT prevalence of 22% (monthly prevalence ranging 9–34%)	
	Tutu et al. [40]	Nov 2005–March 2006	27.7% by microscopy	
	Yatich et al. [37]	Nov–Dec 2006	36.3% by a monoclonal antibody assay	
	Tagbor et al. [41]	March 2007–Sept 2008	16.3% by microscopy at enrolment (parasiste density ˂ 1000 parasites/microlitre 12.1% by microscopy at 36–40 weeks 23% by RDT (for symptomatic women)	
	Asante et al. [47]	2008–2011		38% current or past placental parasitaemia by histology
	Osarfo et al. [42]	July 2011– Oct 2012	12% by combined RDT and microscopy 17% by RDT	28.6% by microscopy (peripheral blood) 23.8% by microscopy (placental blood)
	Asundep et al. [43]	July–August 2011	9% overall using HRP2 assay	
	Ampofo et al. [44]	Sept 2012–April 2014	10.7% by microscopy at study enrolment 6% by microscopy at end of study	
	Dosoo et al. [45]	July 2017–March 2019	20.4% by microscopy	
	Fondjo et al. [32]	July 2018–August 2018	5.5% by microscopy	

### Malaria parasitaemia prevalence

#### Northern savannah zone

Asymptomatic *P. falciparum* peripheral parasitaemia prevalence in pregnant women attending antenatal clinics (ANC) ranged about 50–60% in the 17-year period from 1994 to 2011 with a number of the studies conducted during that time reporting closer to 60% [14–16]. Subsequently, appreciable reductions have been reported from 2013 to 2019 with the highest and lowest measures over this period being 26% and 13.4%, respectively [17–19, 10]. This decline is also reflected in placental parasitaemia prevalence as reports show a reduction from 52% to 2010 to 7% in 2019 [20, 16, 21].

#### Coastal savannah zone

No report of the prevalence of pregnancy-associated malaria infection was found for this zone prior to 2003. In 2003/2004, over a third of women (35.7%) in a study in rural Accra had placental blood parasitaemia on microscopy [22]. Over the period 2003–2010, the reported prevalence of asymptomatic *P. falciparum* infection during pregnancy was generally around 20–28% as detected by peripheral blood film microscopy mostly [22–25] with an isolated report of 5% in an urban setting in Accra, the capital of Ghana [26].

Comparatively, between 2011 and 2019, the prevalence reduced and has ranged between 10 and 17% [27–32] though much lower measures of 3.5–5.5% continue to be recorded in urban and peri-urban areas of Accra [33, 34]. In spite of these relatively lower prevalence recorded for peripheral blood films over the period, polymerase chain reaction (PCR) evaluations showed comparable or much higher prevalence of up to 43% and 12.6% for peripheral and placental parasitaemia, respectively over 2015–2017 [34]. Similarly, close to 50% prevalence of active placental infections, based on histology, among pregnant women presenting for delivery at Korle-Bu Teaching Hospital in Accra over the entire 2017 has been reported [35].

#### Middle transition/forest zone

Studies conducted between 1998 and 2006 in the Ashanti Region reported prevalence of asymptomatic malaria infections during pregnancy around 35% by microscopy [36, 37] while PCR or its combination with microscopy yielded higher measures of up to 63% [38, 39]. One study, however, recorded a relatively lower prevalence of about 27% [40]. At delivery, similar high prevalence measures of 35–60% were observed using placental blood for microscopy, PCR and other methods [39]. Over 2007–2018, an apparent decline was observed with the highest prevalence reported being 19% and the lowest 5% [41–45, 32]. However, within the period, a notably higher prevalence of 24% based on peripheral film microscopy was recorded at delivery in one study conducted in 2011/2012 [42].

In the northern part of this zone (Bono East Region in Fig. 1), prevalence during pregnancy appears to have changed very little comparing the 22% observed by microscopy in 2003/2004 and the 20.4% reported over 2017–2019 [45, 46]. Close to 40% of women in one study conducted over 2008–2010 had placental parasitaemia based on histology [47], but there is no current evaluation to aid comparison.

### Malaria in pregnancy test positivity rates using DHIMS 2 data

**Fig. 3:**
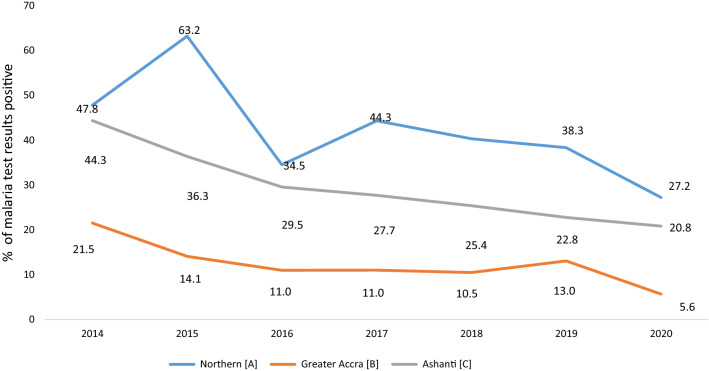
Malaria in pregnancy test positivity rates in Greater Accra, Ashanti and the former Northern Region (2014–2020) (Source: DHIMS 2, April 2021. See Additional file 1: Table S1)

Overall, there was a general decline in MIP test positivity rates (by RDT or microscopy) from 2014 to 2020 with Greater Accra region showing the greatest decline of 74% followed by Ashanti and the former Northern region (now Northern, North-East and Savannah Regions in Fig. 1) which showed reductions by 53% and 43% respectively (see Fig. 3). Within the specified period, Ashanti and the former Northern region, representing the middle transitional/forest zone and northern savannah belts, showed test positivity rates as high as 44% and 63% while Greater Accra, representing the coastal savannah zone, showed much lower rates culminating in a 5.6% test positivity in 2020. Additional file 1: Table S1 shows the numbers of pregnant women with suspected malaria and those tested.

### Factors influencing *P. falciparum* infection and parasite densities in pregnant women

Age, gravidity/parity, gestation, ITN and IPTp-SP use were reported to be key factors influencing malaria parasitaemia and parasite density in pregnancy. Asymptomatic parasite densities reported in Ashanti region between 2007 and 2014 have been mostly below 1000 parasites/microlitre [41, 42, 44] and is similar to recent reports over 2017–2019 from the Brong region [45]. However, a median density of 1720 parasites/microlitre was reported in 2013/2014 in Greater Accra region [29].

Generally, pregnant women of a younger age (less than 20–25 years) were more likely to have parasitaemia or higher parasite densities compared to those above 25–30 years old [15, 24, 30–32, 45]. In the middle forest belt, a significantly higher geometric mean parasite density in pregnant women less than 20 years compared to those above 20 years (333.1/µl vs. 215.4/µl; p = 0.013) was reported [42]. In urban Accra, age had no effect on the occurrence of maternal parasitaemia [33].

Similarly, primi- and secundigravidae had higher odds of parasitaemia and higher parasite densities compared to multigravidae and this was true for both peripheral and placental parasitaemia [15, 21, 30, 32, 38, 41, 45, 47]. In the northern savannah zone, one reported a higher proportion of parasitaemia among primigravidae compared to secundigravidae [16]. However, gravidity/parity did not influence parasitaemia occurrence in urban Accra [33].

The effect of gestation on maternal parasitaemia appeared to be variable. Pregnant women in the third trimester had less likelihood of parasitaemia and high parasite densities compared to those in the earlier stages of pregnancy in the northern savannah zone [15], but other studies did not observe any association between gestation and peripheral parasitaemia or parasite density [30, 33, 42]. It must be noted that the comparison in one of the studies [42] was between second and third trimester women.

The effect of bed net use on maternal parasitaemia was also variable. Some studies reported no association between bed net use and parasitaemia [14, 33] while another observed that parasitaemia was higher among women who did not use ITNs [31]. Intermittent preventive treatment of malaria in pregnancy with SP has been noted to reduce the risk of peripheral parasitaemia including submicroscopic infections [10].

## Discussion

Prevalence of malaria infection in Ghanaian pregnant women, from 1994 to 2019, showed apparent decline across all transmission zones. In the northern savannah zone, the prevalence reduced from about 50–60% over 1994–2010 [14, 15] to about 13–26% for peripheral parasitaemia [17, 10] and ˂ 10% for placental parasitaemia by 2019 [21]. The decline in the middle transition/forest zone was steeper with a reduction also from 60% in the late 1990 s to about 5% in an urban area of the Ashanti Region [32, 38]. Evaluations of malaria infection in pregnancy apparently started much later in the coastal savannah zone with prevalence of up to 28% for peripheral parasitaemia and 35% for placental blood parasitaemia in the period 2003–2010 [22, 23]. This has reduced to about 5% or less in urban and about 11% in peri-urban areas of Accra in the Greater Accra region [32–34].

The apparent decline in asymptomatic parasitaemia prevalence among pregnant women mirrors reported reductions in all-age out-patient malaria cases in Ghana by 57%, under-five malaria admissions and mortality by 46% and 70% respectively between 2005 and 2010 and mid-2015 [48] and falling trends in under-five *P. falciparum* parasite prevalence from 25.5 to 14.1% between 2011 and 2019 [49–52]. This decline also mirrors the reduction in pregnancy-associated malaria test positivity rates from DHIMS 2 data over 2014–2020 though the latter reflects symptomatic cases largely. The review thus provides some evidence that the declining global burden of malaria reflects positively in Ghanaian pregnant women.

Data from current malaria transmission studies in Ghana is lacking but a decline in entomological inoculation rate from about 400 to 139 infective bites/person/year between the early 2000 s and 2014/2015 in the northern zone has been reported [53]. This may underpin the lower prevalence of 14% peripheral parasitaemia and more recently, 7% placental parasitaemia recorded in that zone over 2016–2019 [17, 18, 21]. Similarly, the declining prevalence in the middle transition/forest belt may underlie declining malaria-attributable maternal deaths recorded at the Komfo Anokye Teaching Hospital in Kumasi (Ashanti Region) from 9.4% to 2004 to zero reports over 2017–2020 (KATH Biostatistics Unit, 2005 and 2021) and reflects a 24% reduction in overall malaria deaths in Ghana from 1264 to 2016 to 308 in 2020 [54].

The prevalence of maternal parasitaemia appears to remain relatively higher in the northern savannah transmission zone in spite of the seeming general decline described and this may have to do with the characteristic seasonal malaria transmission pattern, the hot weather that may deter consistent bed net utilization and possibly slower urbanized development compared to Accra in the coastal belt [55, 56]. It has been reported that part of the burden of malaria infection in pregnancy results from pre-conception infection [57] and it is possible this phenomenon may be playing a role in Ghana, especially in the northern zone with marked seasonal transmission. Thus, for the northern savannah belt, it may be time to consider an intervention akin to seasonal malaria chemoprevention that targets the under-fives in women in the reproductive age.

In Greater Accra (Coastal savannah belt), the apparent decline in placental malaria infection prevalence from about 36% to under 1% between 2003/2004 and 2015–2017 [22, 34] could be underlined by a number of factors. The earlier study was in a semi-rural area and the latter in an urban area. Urban development and possibly greater IPTp-SP uptake on account of presumed higher levels of education characteristic of urban settlements [55, 58, 59] may have contributed to the low prevalence in that location even though higher education does not always translate into better IPTp uptake [60]. The means of detection of placental malaria infection could have affected the prevalence reported. The < 1% placental parasitaemia prevalence by blood film microscopy reported in one study [34] contrasts sharply with the almost 50% prevalence by placental histology observed also in urban Accra [35] about the same time as the first study. This reinforces the preference for placental histology in assessing placental parasitaemia [61].

Interestingly, two studies conducted in 2018 and 2019 in urban and rural parts of Greater Accra region respectively reported an approximately equal prevalence of about 11% for peripheral parasitaemia [30, 32]. This suggests that lower transmission in the area could be narrowing rural-urban disparities in asymptomatic malaria infection in pregnancy. Larger studies involving both rural and urban areas in that region would be needed to further explore this observation.

In spite of the generally low prevalence reported in Greater Accra, it must be noted that PCR methods have reported higher prevalence of about 13% and 43% for placental and peripheral parasitaemia respectively even in urban communities [34]. Being more sensitive, PCR methods pick submicroscopic infections and better define the burden of malaria infection in pregnancy. Submicroscopic parasitaemia may underpin the relatively high burden of maternal anaemia and low birthweight in Ghana though the latter appears to be declining [39–42]. In Northern Ghana, however, submicroscopic parasitaemia did not appear to increase the risk of low birthweight [16]. Furthermore, PCR findings of high levels of asymptomatic parasitaemia in pregnant women in Accra [34] may have implications for malaria transmission as asymptomatic infections serve as a reservoir for transmission to mosquitoes [62]. This must be taken into consideration in control efforts by the NMCP as it prepares to usher some districts into pre-elimination by 2025 [54]. Similar high levels of asymptomatic parasitaemia in non-pregnant populations and detected by both rapid diagnostic tests and PCR have been reported in the middle belt [63].

Despite the general decline in parasitaemia in pregnant women, primigravidae, secundigravidae and younger aged women remain most susceptible to infection and this agrees with previous observations elsewhere [64, 65, 57]. Paucigravid women have no or insufficient antibodies to counter placental binding by infected red blood cells expressing specific surface antigens and are thus more likely to have placental infections leading to severe adverse outcomes [66]. Focal interventions may be needed in this group to reduce the burden of parasitaemia. Such interventions could provide other benefits as placental malaria has been linked to pre-eclampsia [35] of which young age is a known risk factor. Intermittent preventive treatment of malaria using dihydroartemisinin-piperaquine (IPTp-DHA-PPQ) may be considered in this sub-population as it was associated with lower incidence of malaria infection during pregnancy and at delivery compared to IPTp-SP [67].

Vaccines to prevent pregnancy-associated malaria is also another option. As the search for an effective candidate continues [68], such vaccines could target adolescent girls before their first pregnancy. Whether these vaccines would be sufficient on their own or need to be supported by additional interventions such as treated nets needs to be explored.

Test positivity rates over 2014–2018 in Ashanti and the former Northern regions, respectively averaging 33% and 46% per year, were appreciably higher than asymptomatic infection prevalence rates observed over the same period. Pregnant women tested in the health facilities are often symptomatic and apparently have higher parasite densities. Asymptomatic malaria infections are less likely to be detected with the conventional light microscopy and rapid diagnostic tests often due to lower densities [69]. The potential for placental sequestration in pregnant women makes detection even more challenging and recommendations have been made to integrate more sensitive molecular methods into research involving detection of asymptomatic malaria infection [69]. Nevertheless, assessing the prevalence of asymptomatic infections in pregnancy using the traditional diagnostic methods can still be relevant for tracking transmission.

The declining trend of malaria infection in pregnancy seems to mirror apparent increments in access to and use of ITNs and IPTp-SP and is consistent with previous reports [8, 10]. Targeted distribution of ITNs to pregnant women has been going on in Ghana since 2004 with a policy of giving bed nets at their first ANC visit. Between 2011 and 2015, over 24 million bed nets were distributed to households in a nationwide exercise [48]. It is likely this may have further benefitted pregnant women and contributed to the increased ownership reported in studies over 2011–2019 [45, 52, 70–72].

Similarly, bed net utilization has also improved over the years [70, 72] but still continues to lag substantially behind ownership. Various reasons including the hot weather, preference for fans, insecticide sprays or coils, saving the net for later use or repurposing the net for other uses, such as fencing gardens have been given for this observation [52, 56]. A higher utilization of up to 95% recorded in 2017 in Navrongo (Upper East region) in the northern savannah zone [72] could be linked to years of research activity in the area by the Navrongo Health Research Centre leading to the pregnant women probably getting more accustomed to ITN use. This has, however, not translated into substantial decrease in malaria parasitaemia in pregnancy in the zone.

In spite of the recent successes, ITN coverage has been less than optimal and ownership has been plagued by health system challenges including frequent stock-outs [56]. Knowledge gaps relating to malaria transmission or a lack of appreciation of the adverse outcomes of malaria in pregnancy may also underlie reduced utilization [73]. It may be beneficial to review the method of exchange of malaria information at ANC clinics to include more engaging methods such as video/graphic illustrations of the pathophysiology underlying placental sequestration and the development of its adverse outcomes with a narrative in various local languages. Adults learn best when teaching strategies incorporate visual, auditory and other approaches that emphasize more active participation [74].

Similar to ITN ownership and use, coverage of three or more doses of SP for IPTp in Ghana, per current WHO recommendation [75], has increased from 27% to 2008 to 83% in 2020 [52,54, 76, 77] and contributed to parasitaemia reduction [10]. National coverage for ≥ 4 doses in 2020 was up to 38% [54].

Although of contextual value only to this review which focused on asymptomatic parasitaemia prevalence from field studies, using DHIMS 2 data for only 5 out of 16 regions gives a restricted view of MIP test positivity rates in Ghana and is a limitation. Secondly, the contribution of changing rainfall and temperature patterns, over the review period, was not assessed. The findings, however, are relevant and draw attention to gains made and weaknesses that need to be addressed to consolidate and improve the gains. It must be remembered that Ghana is part of the high burden high impact countries for malaria and the findings are important to help reduce the malaria burden, especially in pregnant women.

## Conclusions

There has been an appreciable decline in malaria parasite prevalence in Ghanaian pregnant women over the last 25 years similar to trends observed in children under-five in Ghana and globally. This development appears to be aligned with increased access to ITNs and uptake of at least 3 doses of IPTp-SP. The greatest declines appear to be in urban areas and reinforce known disparities between rural and urban parasite burdens. Despite this decline, submicroscopic parasitaemia remains a risk and together with asymptomatic parasitaemia, pose challenges for reducing transmission as part of control efforts. Larger-scale assessments of the local burden of submicroscopic parasitaemia and how they relate to pregnancy outcomes are needed. Additionally, further studies are needed to assess the impact of the observed reduction in asymptomatic malaria parasite prevalence in Ghanaian pregnant women on the burden/trends in maternal anaemia, low birthweight, stillbirth and other adverse outcomes of MIP.

## Supplementary Information

**Additional file 1: Table S1.** Numbers of pregnant women with malaria from 2014 to 2020 in Ashanti Region, Greater Accra and the former Northern Regions.

## Data Availability

All data generated or analysed during this study are included in this published article and its Additional files.

## Abbreviations

MIP: Malaria in pregnancy
ITN: Insecticide-treated net
IPTp-SP: Intermittent preventive treatment of malaria in pregnancy with sulfadoxine–pyrimethamine
DHIMS 2: District health information management system 2
MPH: Masters in Public Health
KATH: Komfo Anokye Teaching Hospital
PCR: Polymerase chain reaction
DHA-PPQ: Dihydroartemisinin–piperaquine
NMCP: National Malaria Control Programme
