# Supplementary material for: Trends of malaria infection in pregnancy in Ghana over the past two decades: a review
Source: Malar J. 2022 Jan 4;21:3. doi: 10.1186/s12936-021-04031-3 (PMC8725495; doi:10.1186/s12936-021-04031-3)
Supplement: Supplementary file 1 — Additional file 1: Table S1. Numbers of pregnant women with malaria from 2014 to 2020 in Ashanti Region, Greater Accra and the former Northern Regions. [file 12936_2021_4031_MOESM1_ESM.docx]

**Additional file 1**

**Table S1: Numbers of pregnant women with malaria from 2014 to 2020 in Ashanti Region, Greater Accra and the former Northern Regions**

| **Region** | **Year** | **Number of suspected uncomplicated MIP tested** | **Number of suspected MIP testing positive** | **% positive** |
| --- | --- | --- | --- | --- |
| **ASHANTI** | 2014 | 16,617 | 7,369 | 44.3 |
|  | 2015 | 21,861 | 7,945 | 36.3 |
|  | 2016 | 33,185 | 9,804 | 29.6 |
|  | 2017 | 32,664 | 9,042 | 27.7 |
|  | 2018 | 37,327 | 9,473 | 25.4 |
|  | 2019 | 34,196 | 7,784 | 22.8 |
|  | 2020 | 35, 575 | 7403 | 20.8 |
|  |  |  |  |  |
| **GREATER ACCRA** | 2014 | 12,094 | 2,602 | 21.5 |
|  | 2015 | 15,960 | 2,247 | 14.1 |
|  | 2016 | 23,954 | 2,627 | 11.0 |
|  | 2017 | 19,428 | 2,137 | 11.0 |
|  | 2018 | 17,073 | 1,786 | 10.5 |
|  | 2019 | 20,460 | 2,670 | 13.1 |
|  | 2020 | 25,125 | 1,418 | 5.6 |
|  |  |  |  |  |
| ^&^**NORTHERN** (former) | 2014 | 7,653 | 3,663 | 47.8 |
|  | 2015 | 7,014 | 4,431 | 63.2 |
|  | 2016 | 8,586 | 2,965 | 34.5 |
|  | 2017 | 8,029 | 5,555 | 44.3 |
|  | 2018 | 8,656 | 3,491 | 40.3 |
|  | 2019 | 11,905 | 4,559 | 38.3 |
|  | 2020 | 20,757 | 5,640 | 27.2 |

**Source**: DHIMS 2 Data (accessed in 2021). ^&^Data for the former Northern region was obtained by summing up the respective data for the present Northern, North-East and Savannah regions.
